# Role of hepatic and portal venous Doppler in constrictive pericarditis: insights from a case series

**DOI:** 10.1093/ehjcr/ytag096

**Published:** 2026-02-04

**Authors:** Nagarjuna Panidapu, Praveen Kumar Neema, Jes Jose, Devika Poduval, Don Jose Palamattam, Divya Jacob, Thushara Madathil, Praveen Kerala Varma

**Affiliations:** Department of Cardiac Anesthesia, Amrita Institute of Medical Sciences, Kochi 682041, India; Department of Cardiac Anesthesia, Amrita Institute of Medical Sciences, Kochi 682041, India; Department of Cardiac Anesthesia, Amrita Institute of Medical Sciences, Kochi 682041, India; Department of Cardiac Anesthesia, Amrita Institute of Medical Sciences, Kochi 682041, India; Department of Cardiac Anesthesia, Amrita Institute of Medical Sciences, Kochi 682041, India; Department of Cardiac Anesthesia, Amrita Institute of Medical Sciences, Kochi 682041, India; Department of Cardiac Anesthesia, Amrita Institute of Medical Sciences, Kochi 682041, India; Department of Cardiac Surgery, Amrita Institute of Medical Sciences, Kochi 682041, India

**Keywords:** Constrictive pericarditis, Hepatic venous Doppler, Portal venous Doppler, Echocardiography, VExUS, Case series

## Abstract

**Background:**

Constrictive pericarditis is characterized by diffuse thickening, fibrosis, and calcification of the pericardium, leading to diastolic dysfunction and heart failure. Echocardiographic assessment typically focuses on *trans*-mitral and *trans*-tricuspid inflows as well as venous flows in the superior vena cava, pulmonary veins, and hepatic veins. The diagnostic and perioperative utility of hepatic and portal venous Doppler assessment in constrictive pericarditis, however, remains insufficiently explored.

**Case summary:**

We present a case series of patients with constrictive pericarditis in whom portal and hepatic venous Doppler interrogation was performed alongside conventional echocardiographic evaluation. The Doppler findings were integrated into the perioperative management strategy, contributing to a comprehensive understanding of venous return dynamics in these patients.

**Discussion:**

Our experience highlights the potential role of portal and hepatic venous Doppler assessment in the perioperative evaluation of constrictive pericarditis. These parameters may provide incremental value by complementing standard echocardiographic indices, thereby offering additional haemodynamic insights and facilitate tailored perioperative management. Further studies are warranted to validate their diagnostic and prognostic significance in this context.

Learning points

- Hepatic vein Doppler A/D ratio ≥ 0.8 suggests impaired right ventricular filling in constrictive pericarditis
- Portal vein pulsatility index ≥ 50% preoperatively indicates severe venous congestion; ≤ 30% postoperatively signifies effective decongestion
- Normalization of both parameters after pericardiectomy may help predict an uneventful postoperative course
- Persistence of abnormal Doppler parameters despite normalized central venous pressure may identify patients at risk for postoperative complications

## Introduction

Constrictive pericarditis (CP) is a disease of the pericardium, characterized by diffuse thickening, fibrosis, and calcification of the pericardium, leading to diastolic heart failure. As the disease process advances, the impaired ventricular filling progresses to a stage of low cardiac output state, systemic venous congestion, pedal oedema, hepatic congestion, and ascites.^1^ The initial diagnosis of CP is usually made using 2-dimensional echocardiography, which include thickened and calcific pericardium, dilated atria, interventricular septal bounce (exaggerated ventricular interdependence), and plethoric superior and inferior vena cavae.^1,2^ The other classical description is an exaggerated increase in *trans*-tricuspid inflow and a simultaneous decrease in *trans*-mitral inflow with inspiration due to dissociation of intraventricular and intrathoracic pressures and increased interventricular dependence.^2^

Doppler interrogation of hepatic and portal veins is conventionally included for the assessment of systemic venous filling and part of vascular excess ultrasound (VExUS) grading. Multiple reports have shown the utility of hepatic venous Doppler flow (HVDF) in patients with CP.^3–5^ Portal vein pulsatility is another echocardiographic indicator for diagnosing right ventricular dysfunction.^6^ Gonzalez *et al*.^5^ described the utility of portal vein pulsatility in the intraoperative period in patients undergoing pericardiectomy. However, there are limited reports on the combined usage of hepatic and portal venous Doppler flow profiles in the perioperative period. In this case series, we describe the perioperative evolution of the flow profiles in hepatic and portal veins before and after the release of pericardial constriction and how they helped in titrating the perioperative management and improved the outcomes. Patient consent was obtained to report the perioperative images and data.

## Summary figure

**Figure:**
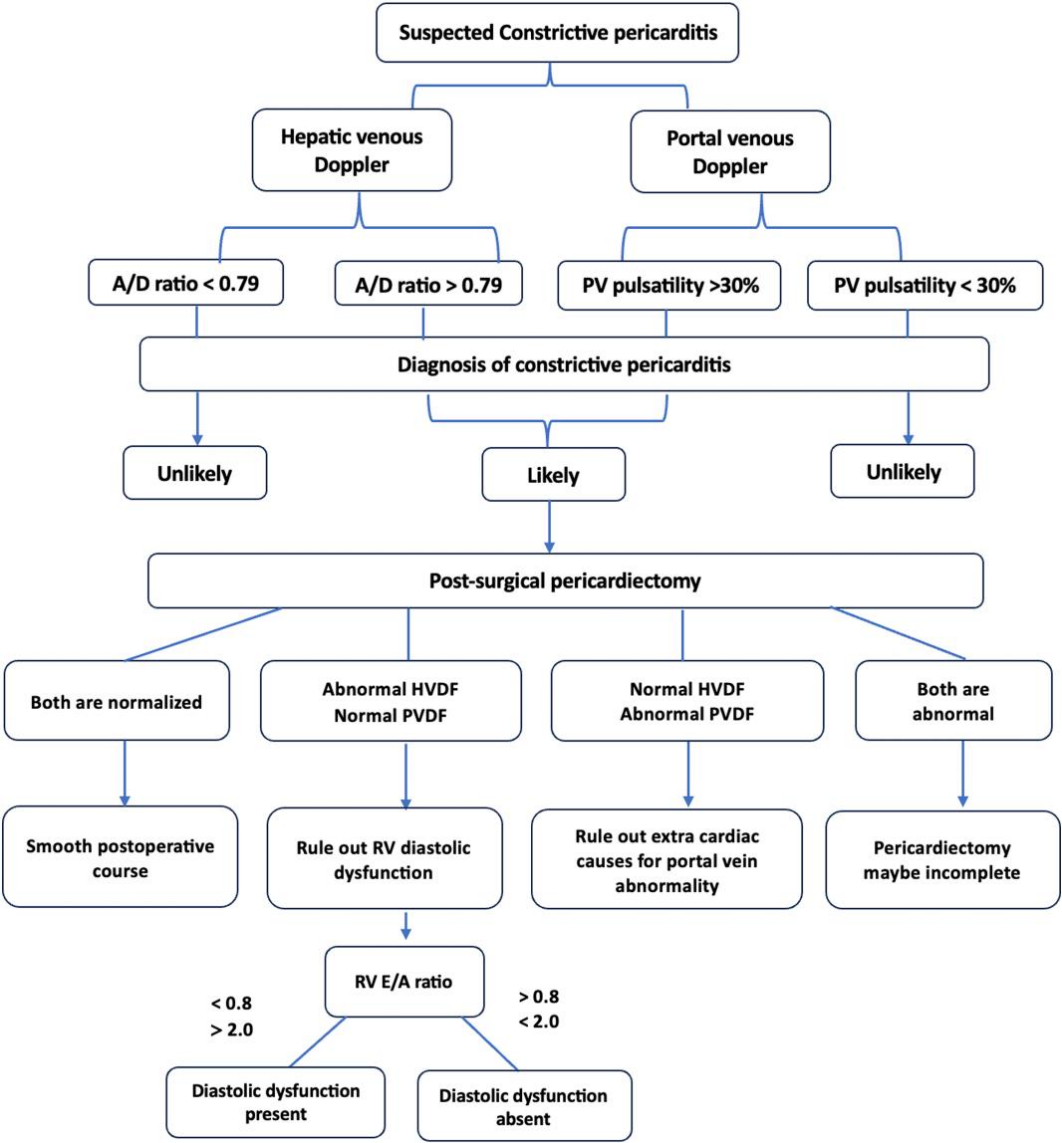


## Case details

### Case 1

A 55-year-old male presented with progressive dyspnoea, pedal oedema, and increasing abdominal girth over two months. There was no other significant past medical history. Preoperative transthoracic echocardiography (TTE) revealed features consistent with CP. Computed tomography of the chest demonstrated a diffusely calcified pericardium. The patient was electively scheduled for pericardiectomy.

**Figure 1:**
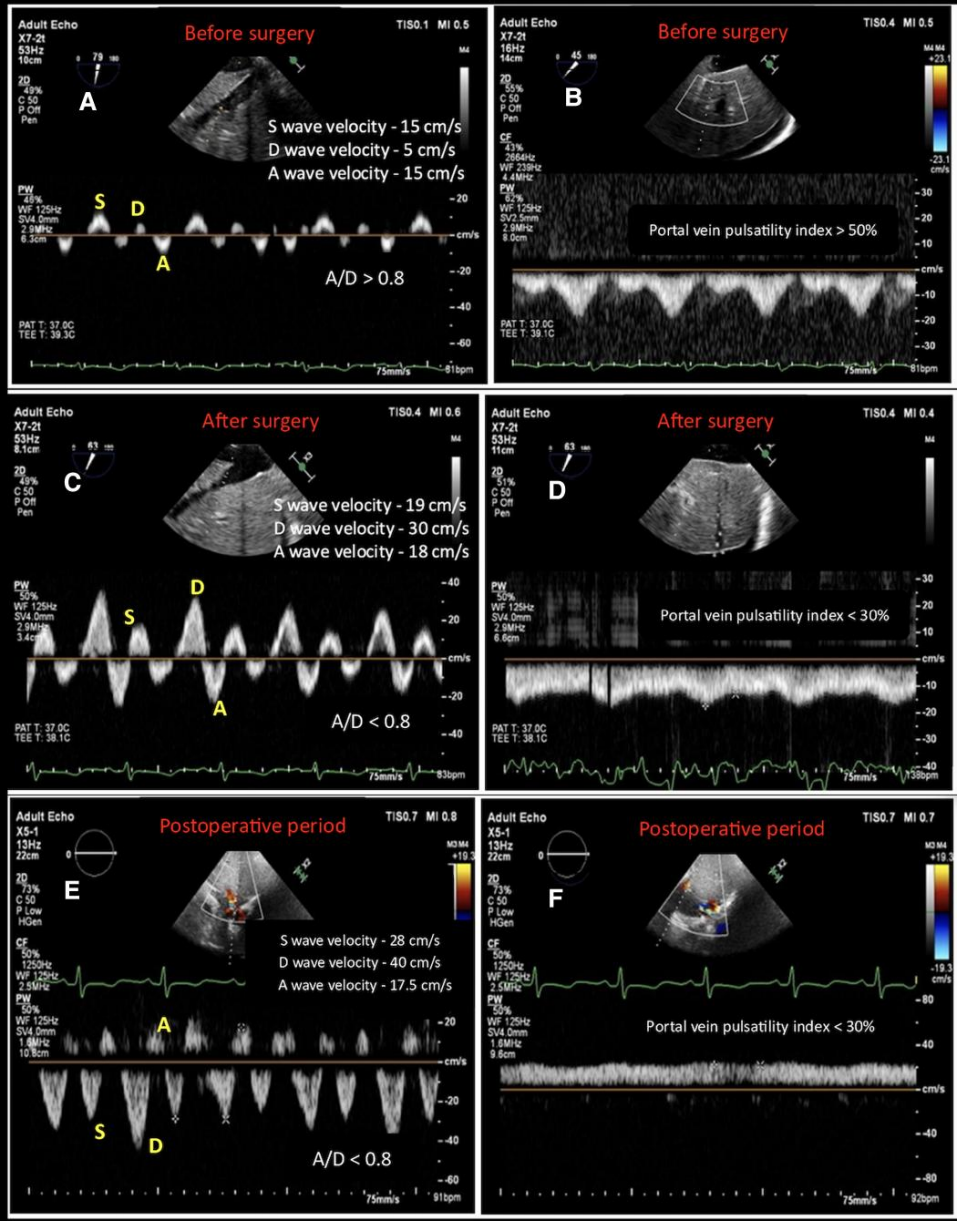
Hepatic venous Doppler waveforms (*A*, *C*, *E*) and portal venous Doppler waveforms (*B*, *D*, *F*) are shown at three perioperative stages. (*A*) and (*B*) illustrate the preoperative findings obtained by transoesophageal echocardiography. (*C*) and (*D*) present the Doppler profiles immediately after pericardiectomy. (*E*) and (*F*) display the postoperative venous Doppler waveforms acquired by transthoracic echocardiography on follow-up.

In the operating room, haemodynamic monitoring revealed markedly elevated central venous pressure (CVP) of 35 mm Hg, pulmonary arterial pressures of 75/44 mm Hg, and a depressed cardiac index of 0.9 L/min/m^2^. A transoesophageal echocardiography (TEE) probe was introduced, which confirmed preoperative TTE findings. Additionally, spontaneous echo contrast was noted in the inferior vena cava. HVDF demonstrated very low velocities of the systolic (S), diastolic (D), and retrograde atrial (A) waves, and A wave greater than D wave (A/D > 0.80) (*Figure 1A*). Portal vein Doppler flow (PVDF) showed a pulsatile waveform mimicking the S- and D- pattern of hepatic venous flow, with a portal vein pulsatility index > 50% (*maximal velocity—minimal velocity/maximal velocity; normal < 30%*) (*Figure 1B*). The patient underwent pericardiectomy uneventfully. By the end of surgery, CVP had reduced to 16 mm Hg and cardiac index increased to 2.0–2.3 L/min/m^2^. HVDF interrogation demonstrated increased velocities of S, D, and A waves, though with an S/D ratio < 1, while A/D became < 0.80 (*Figure 1C*). PVDF showed reduced pulsatility with a pulsatility index of < 30% (*Figure 1D*). Right ventricular systolic function was preserved; however, *trans*-tricuspid E/A ratio was 0.7, consistent with diastolic dysfunction, warranting initiation of milrinone infusion. The intraoperative period was otherwise uneventful. The patient was transferred to the intensive care unit (ICU) and extubated on postoperative Day 1. In the ICU, fluid resuscitation and milrinone infusion were continued. On the second postoperative day, HVDF showed a further increase in S and D velocities, however, S/D remained < 0.8 (*Figure 1E*) and a continuous PVDF pattern with pulsatility index < 30% (*Figure 1F*). The remainder of the postoperative course was uneventful, and the patient was discharged to the ward on third postoperative day.

### Case 2

A 50-year-old man, with a history of tuberculosis one year prior, was admitted with complaints of exertional dyspnoea and palpitations. Pre-operative electrocardiography revealed low-voltage QRS complexes, and a lateral chest X-ray demonstrated circumferential pericardial calcification surrounding the ventricles. Cardiac catheterization showed a characteristic ‘square-root’ sign, confirming the diagnosis of CP. The patient was referred for surgical pericardiectomy.

**Figure 2:**
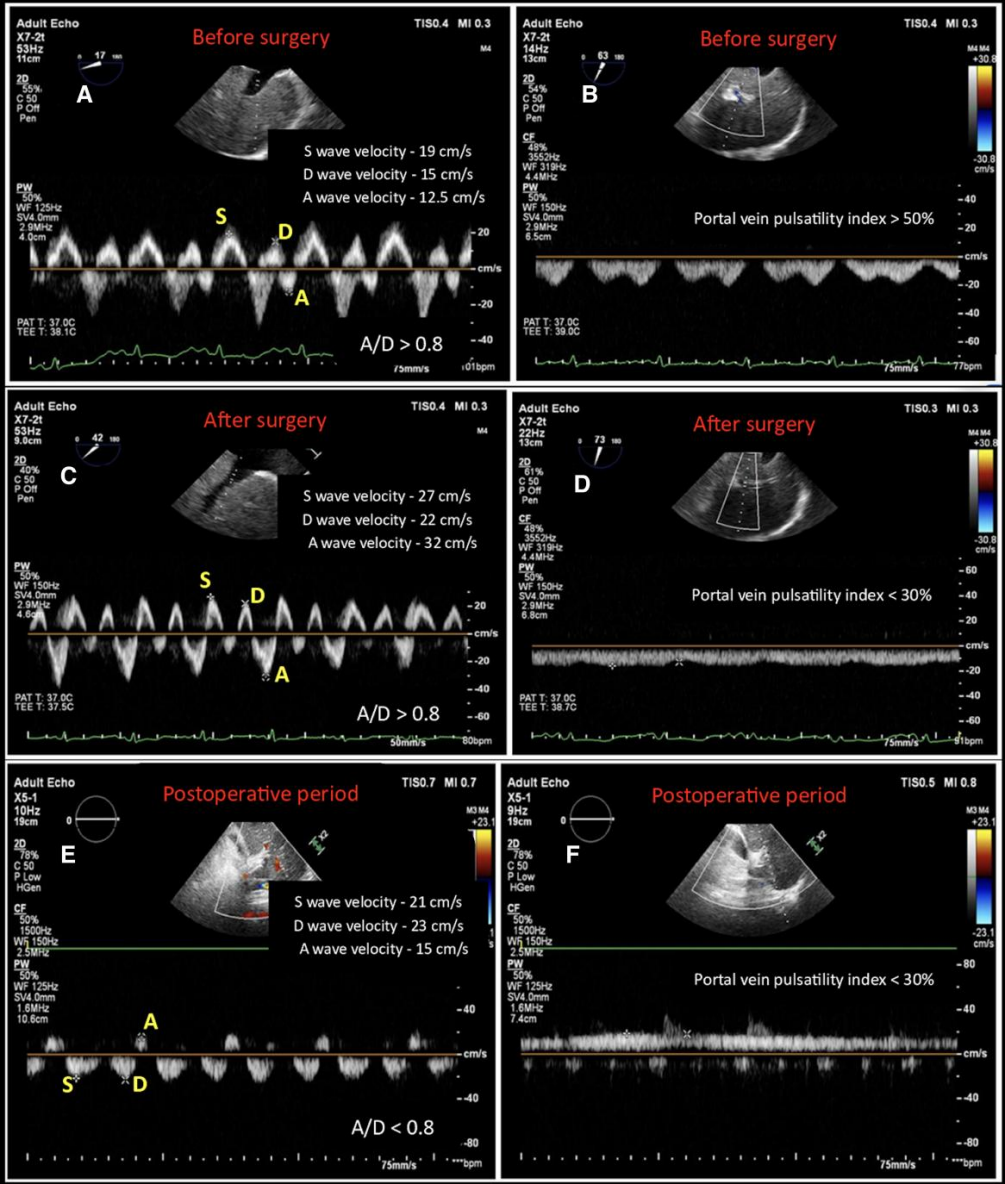
Hepatic venous Doppler waveforms (*A*, *C*, *E*) and portal venous Doppler waveforms (*B*, *D*, *F*) are shown at three perioperative stages. (*A*) and (*B*) illustrate the preoperative findings obtained by transoesophageal echocardiography. (*C*) and (*D*) present the Doppler profiles immediately after pericardiectomy. (*E*) and (*F*) display the postoperative venous Doppler waveforms acquired by transthoracic echocardiography on follow-up.

In the operating room, haemodynamic parameters before surgical incision were: CVP of 20 mm Hg, pulmonary arterial pressures of 45/23 mm Hg, and a cardiac index of 1.3 L/min/m^2^. TEE revealed a dilated inferior vena cava (30 mm) and hepatic veins (17 mm) with spontaneous echo contrast. Preoperatively, HVDF showed blunted systolic and diastolic waves, with increased A-wave velocity (A/D > 0.80) (*Figure 2A*). PVDF showed S- and D-pattern with a pulsatility index > 50% (*Figure 2B*). The patient underwent extensive pericardiectomy. After surgery, HVDF showed increased S, D, and A velocities, with a nearly three-fold rise in A-wave velocity (A/D > 0.80) (*Figure 2C*), and PVDF demonstrated continuous flow with a pulsatility index < 30% (*Figure 2D*). Airway pressures increased from 16 to 29 cm of water by the end of surgery. A bolus of intravenous furosemide (40 mg) was administered, followed by a continuous infusion at 2 mg/h. By the end of surgery, CVP had decreased to 12 mm Hg, and the cardiac index improved to 2.2–2.5 L/min/m^2^. The remainder of the intraoperative course was uneventful.

After transfer to the ICU, lung ultrasound demonstrated a B-profile. Dopamine infusion was initiated at 2.5 mcg/kg/min, and the furosemide infusion was increased to 4 mg/h. On the first postoperative day, the patient was extubated to non-invasive ventilation. CVP was reduced to 5–8 mm Hg and the cardiac index remained elevated (3.5–4.0 L/min/m^2^). TTE examination on second postoperative day showed improved HVDF, with increased S, D, and A waves (A/D < 0.79) (*Figure 2E*) and normal continuous PVDF (pulsatility index < 30%) (*Figure 2F*). Despite these improvements, the patient developed postoperative acute kidney injury and delirium. He was discharged from ICU on fifth postoperative day.

### Case 3

A 35-year-old man with a known history of Hodgkin’s lymphoma was admitted with dyspnoea, pedal oedema, and ascites. He had been diagnosed with CP at another hospital and was referred to our centre for pericardiectomy.

**Figure 3:**
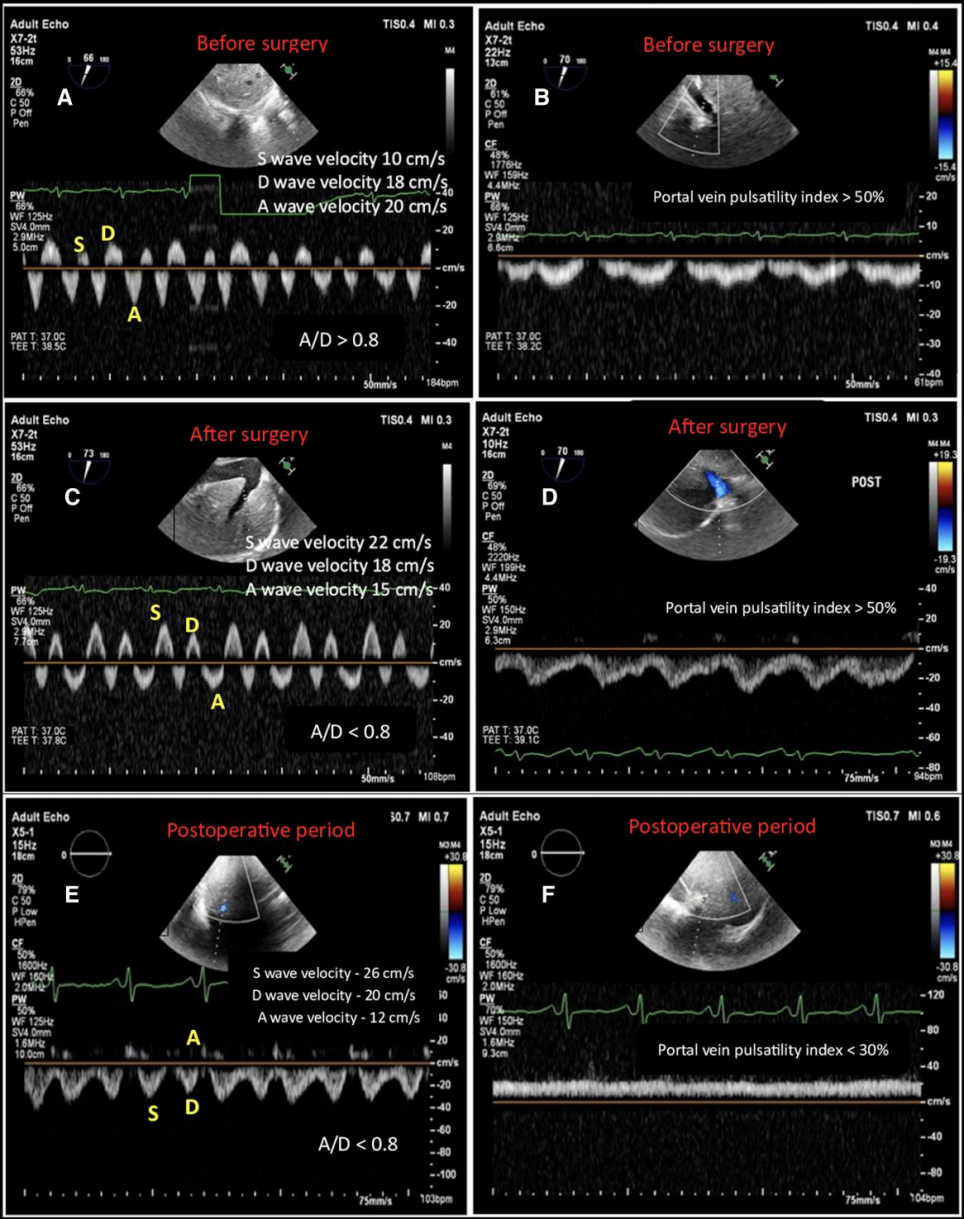
Hepatic venous Doppler waveforms (*A*, *C*, *E*) and portal venous Doppler waveforms (*B*, *D*, *F*) are shown at three perioperative stages. (*A*) and (*B*) illustrate the preoperative findings obtained by transoesophageal echocardiography. (*C*) and (*D*) present the Doppler profiles immediately after pericardiectomy. (*E*) and (*F*) display the postoperative venous Doppler waveforms acquired by transthoracic echocardiography on follow-up.

In the operating room, haemodynamic parameters revealed a CVP of 34 mm Hg, pulmonary arterial pressures of 37/24 mm Hg, and a cardiac index of 0.9 L/min/m². Preoperative HVDF demonstrated very low S and D -velocities, with an elevated A/D ratio (> 0.80) (*Figure 3A*). PVDF showed an S- and D-wave pattern with a pulsatility index > 50% (*Figure 3B*). After pericardiectomy HVDF normalized (*Figure 3C*), while PVDF continued to remain abnormal, with a pulsatility index > 50% (*Figure 3D*). Airway pressures remained within normal limits. By the end of surgery, CVP had decreased to 10 mmHg, and the cardiac index improved to 1.6 L/min/m². Following transfer to the ICU, TTE confirmed persistence of the abnormal PVDF pulsatility index (> 50%), while HVDF remained normal. Significant ascites was also noted, and 1500 mL of ascitic fluid was drained. On the second postoperative day, HVDF showed normal flow (A/D ratio < 0.80) (*Figure 3E*), and PVDF improved with a pulsatility index < 30% (*Figure 3F*). The remainder of the postoperative course was uneventful, and the patient was discharged to the ward on third postoperative day.

## Discussion

CP remains a diagnostic challenge due to its overlapping clinical, haemodynamic, and echocardiographic features with restrictive cardiomyopathy. While invasive cardiac catheterization continues to be regarded as gold standard for diagnosis, non-invasive modalities play an increasingly important role in early recognition and perioperative assessment. In the present case series, we highlight the perioperative utility of HVDF and PVDF patterns in patients undergoing pericardiectomy for CP. These Doppler-derived indices not only correlated with elevated filling pressures and systemic venous congestion preoperatively, but also demonstrated dynamic changes following surgical pericardiectomy. Our observations underscore their role as sensitive markers of haemodynamic improvement, particularly when conventional indices maybe equivocal.

**Figure 4:**
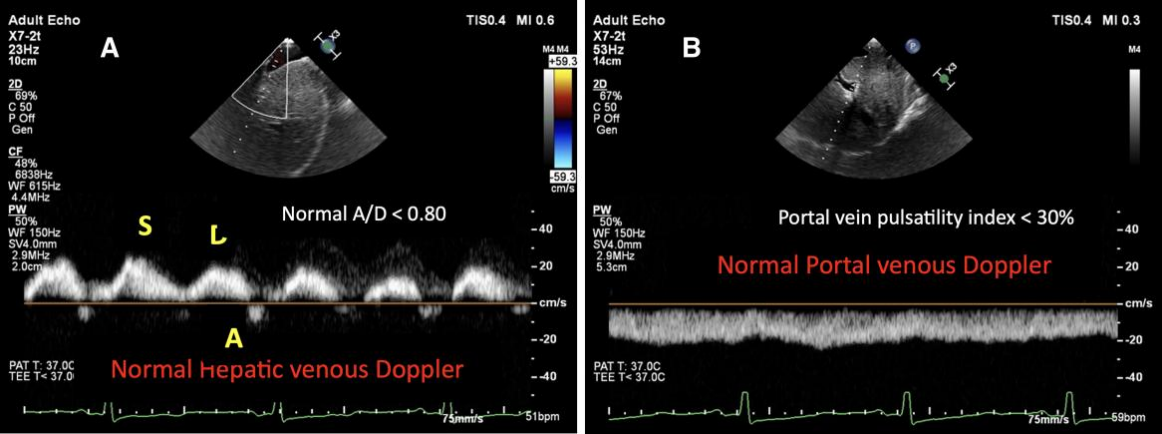
Normal hepatic venous Doppler waveform shows S, D, and a waves (panel *A*) (TEE image); normal portal venous Doppler shows continuous waveform with no pulsatility (panel *B*) (TEE image).

In normal healthy individuals, HVDF exhibits a triphasic waveform consisting of two antegrade components, the systolic (S) and diastolic (D) waves, and a brief retrograde atrial (A) wave. The S wave predominates (S/D > 1) and reflects normal right-sided compliance and filling. The normal hepatic venous A/D ratio is < 0.80, indicating that the A wave is smaller than the diastolic forward component, consistent with unobstructed right atrial filling. In contrast, PVDF is typically continuous and monophasic, directed hepatopetally with minimal pulsatility (pulsatility index < 30%) (*Figure 4*). These patterns become distorted when right atrial or pericardial pressures rise. CP alters ventricular filling through a non-compliant pericardial shell, producing an exaggerated ventricular interdependence and dissociation of intracardiac from intrathoracic pressures. This physiology drives characteristic Doppler signatures, especially the systemic veins that can complement standard echocardiography and even invasive catheterization.^1,7^ In CP, hepatic vein typically shows prominent expiratory diastolic flow reversal due to abrupt cessation of right ventricular filling when pericardial constraint shifts the septum and raises right-sided diastolic pressures. Respiratory dependence of venous return amplifies these changes. Contemporary guidance highlights end-expiratory hepatic vein flow A/D > 0.8 as supportive of CP.^1,8,9^ In this case series, all patients demonstrated a preoperative HVDF A/D ratio > 0.8 (*Table 1*). Following pericardiectomy, the A/D ratio normalized (< 0.80) in the first and third patients, but remained > 0.80 in the second patient. In contrast, CVP decreased significantly in all three patients, reaching near-normal levels. Traditionally, normalization of CVP has been regarded as an indicator of adequate pericardiectomy, while few studies stood against this parameter.^10^ However, despite CVP normalization, the second patient experienced a stormy postoperative course. Notably, this was the only patient with persistent elevation of the hepatic venous A/D ratio after surgery, suggesting that HVDF may provide additional prognostic value and help identify patients at risk of adverse outcomes despite apparently adequate surgical decompression. In addition, we have evaluated PVDF profiles with the evolution of venous congestion and how their relation changed after pericardiectomy. In our first two cases, markedly abnormal preoperative high portal vein pulsatility (> 50%) improved immediately after pericardiectomy, with portal vein showing near continuous flow. This temporal resolution mirrors classic CP physiology and relief of pericardial constraint. However, in the third case, portal vein pulsatility remained > 50% until ascitic decompression, and then improved by second postoperative day (*Table 1*). This highlights the splanchnic capacitance and inertia of portal venous hemodynamics, often slower to normalize than hepatic venous signals, and influenced by extra pericardial factors such as ascites and hepatic sinusoidal pressure.

**Table 1:** Patient-wise echocardiographic and haemodynamic profile before and after surgery

		Patient 1	Patient 2	Patient 3
**Central venous pressure (mm Hg)**	Preop	35	20	34
	After surgery	16	12	10
**Cardiac index (L/min/m^2^)**	Preop	0.9	1.3	0.9
	After surgery	2–2.3	2.2–2.5	1.6
**Hepatic vein A/D ratio**	Preop	> 0.8	> 0.8	> 0.8
	After surgery	< 0.8	> 0.8	< 0.8
**Portal vein pulsatility index**	Preop	> 50%	> 50%	> 50%
	After surgery	< 30%	< 30%	> 50%
**Additional findings**		Right ventricular E/A 0.7	High airway pressures	Abdominal ascites
			B-profile in lung ultrasound	
**Interventions**		Milrinone	Diuretics	Ascitic fluid tapping
			Dopamine	
**Changes after interventions**		Right ventricular diastolic dysfunction persisted	Hepatic vein A/D < 0.8	Portal vein pulsatility < 30%
**Postoperative course**		Uneventful	Acute kidney injury	Uneventful
			Non-invasive ventilation	
			Delirium	

Right ventricular dysfunction is one of the early postoperative complications after pericardiectomy. The proposed mechanisms for right ventricular failure include atrophy due to prolonged constriction, systolic dysfunction, increased venous return to right heart, and pulmonary thromboembolism after pericardiectomy.^11–13^ In our first patient, right ventricular systolic parameters were within normal limits after surgery, ruling out the possibility of RV systolic dysfunction and pulmonary thromboembolism. Although tricuspid inflow E/A can be tracked after pericardiectomy, current literature and guidance do not support *trans*-tricuspid E/A as a stand-alone marker of postoperative right ventricular diastolic function in CP. Instead, reduction in the respiratory variation of tricuspid E wave, evolution of annular e′/E′ patterns, and hepatic-vein Doppler normalization better reflect physiologic release and have greater face validity in this setting.^14–17^ In our first patient, *trans*-tricuspid E/A was 0.7 and hepatic vein S/D < 1 after surgery, suggesting a possibility of right ventricular diastolic dysfunction, warranted an infusion of milrinone. Similarly, in our second patient, an acute increase in right ventricular preload likely occurred following pericardiectomy, leading to pulmonary congestion, rising airway pressures, and the subsequent need for non-invasive ventilation in the postoperative period. With ongoing diuresis, pulmonary congestion gradually resolved. However, the VExUS score remained elevated, suggesting persistent systemic venous congestion, which may have contributed to the development of acute kidney injury and postoperative delirium.

Positive-pressure ventilation modifies venous return and can attenuate Doppler features.^18^ HVDF can demonstrate improvement with effective pericardial decompression, but readers should interpret values in the context of altered airway pressures and intrathoracic pressure swings.^3^ While portal vein pulsatility index has its roots in hepatology, it has emerged as a robust marker of systemic venous congestion and adverse outcomes in cardiac patients, with pulsatility index > 50% was shown be associated with high right-sided pressures and organ congestion.^19,20^ Our series extends these insights into CP perioperatively, showing pulsatility index can track decongestion after pericardiectomy and it can normalize later than HVDF when splanchnic congestion or ascites persist.

## Conclusion

While the guidelines emphasize on multimodal imaging and invasive hemodynamics in CP patients, incorporating HVDF and PVDF in this framework maybe a plausible option, offering a distinct advantage over reliance on normalized CVP. Unlike CVP, which may normalize despite residual haemodynamic compromise, HVDF and PVDF provide dynamic, physiology-rich data that sensitively reflect venous congestion and right-sided filling pressures. These Doppler parameters can serve as valuable additions to the echocardiographers armamentarium for intraoperative decision-making and postoperative decongestive therapy. Larger prospective studies are warranted to validate these findings and to define their role within perioperative echocardiographic algorithms.

## Lead author biography


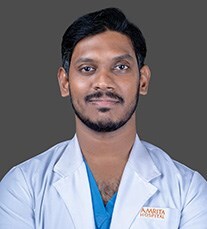


Nagarjuna Panidapu is an Assistant Professor in the Department of Cardiac Anesthesia at Amrita Institute of Medical Sciences, Kochi, India. His clinical and academic interests include peri-operative echocardiography, advanced haemodynamic monitoring, and research in cardiac anaesthesia. He has authored multiple peer-reviewed publications and case reports focusing on intra-operative echocardiographic assessment, valvular heart disease, and peri-operative optimization strategies.

## Notes

**Consent:** Informed consent was obtained from the patients to publish the details and use echocardiographic-related information. Informed consent was obtained from the patients in line with COPE guidance.

**Funding:** None declared.

**Data availability:** The data underlying this article will be available upon requesting the corresponding author.
